# The Role of Tim-3 on dNK Cells Dysfunction During Abnormal Pregnancy With *Toxoplasma gondii* Infection

**DOI:** 10.3389/fcimb.2021.587150

**Published:** 2021-02-26

**Authors:** Teng Li, Lijun Cui, Xiaoyan Xu, Haixia Zhang, Yuzhu Jiang, Liqin Ren, Chunyan Yang, Xianbing Liu, Xuemei Hu

**Affiliations:** ^1^Department of Immunology, Binzhou Medical University, Yantai, China; ^2^Medicine & Pharmacy Research Center, Binzhou Medical University, Yantai, China; ^3^School of Stomatology, Binzhou Medical University, Yantai, China

**Keywords:** *Toxoplasma gondii*, dNK cells, Tim-3, inhibitory molecule, abnormal pregnancy outcomes

## Abstract

Vertical transmission of *Toxoplasma gondii* (*T. gondii*) infection during gestation can result in severe complications such as abortion, congenital malformation, fetal teratogenesis, etc. Immune inhibitory molecule Tim-3 was discovered to be expressed on some decidual immune cells and participates in the maintenance of maternal-fetal tolerance. Dysregulation of Tim-3 expression on decidual NK (dNK) cells was observed in several cases of pregnancy complications, whereas the role of Tim-3 on dNK cells during *T. gondii* infection remains unclear. In the present study, *T. gondii* infected Tim-3^-/-^ pregnant mice, and anti-Tim-3 neutralizing antibody treated and infected human dNK cells were successfully established to explore the role of Tim-3 in dysfunction of dNK cells during abnormal pregnancy. Our results illustrated that Tim-3^-/-^ pregnant mice displayed more worse pregnancy outcomes with *T. gondii* infection compared to infected WT pregnant mice. Also, it demonstrated that Tim-3 expression on dNK cells was significantly down-regulated following *T. gondii* infection. Data suggested a remarkable activation of dNK cells in Tim-3^-/-^ mice and anti-Tim-3 neutralizing antibody treated and infected groups, with higher ratios of activating receptor NKG2D to inhibitory receptor NKG2A or KIR2DL4, IFN-γ/IL-10, and increased granule production compared with that of the infected group. Mechanism analysis proved that *T. gondii-*induced Tim-3 down-regulation significantly activated the phosphatidylinositol-3-kinase (PI3K)-AKT and JAK-STAT signaling pathway, by which the GranzymeB, Perforin, IFN-γ, and IL-10 production were further up-regulated. Our research demonstrated that the decrease of Tim-3 on dNK cells caused by *T. gondii* infection further led to dNK cells function disorder, which finally contributed to the development of abnormal pregnancy outcomes.

## Introduction

*Toxoplasma gondii* (*T. gondii*) is an obligate intracellular parasite which is capable of infecting all species of warm-blooded animals *via* the ingestion of tissue cysts or sporulated oocysts (Robert-Gangneux and Dardé, 2012). Although *T. gondii* can be controlled in subjects with healthy immune systems, it can cause life-threatening damage for patients with immunocompromised states (Yarovinsky, 2014). As a pathogen of human TORCH syndrome (toxoplasmosis, rubella, cytomegalovirus, herpes simplex, and other agents), the vertical transmission of *T. gondii* from an infected mother to her fetus can lead to devastating consequences such as pregnancy loss or severe disease in the neonate, including blindness, developmental delay, or neurological manifestations (Arora et al., 2017). Successful reproduction depends on the homeostasis of maternal-fetal tolerance (Mold and McCune, 2012). The specialized immune microenvironment is sustained by the dynamic orchestration of immune cells (dNK cells, macrophages and Treg cells), cytokines and enzymatic factors (Erlebacher, 2013). In the first trimester of gestation, dNK cells represent the largest population, comprising 70% of decidual immune cells (Trundley and Moffett, 2004). The dNK cells modulate maternal-fetal tolerance by maintaining stable functional molecule expression (NKG2A and KIR2DL4) and cytokines production (Rouas-Freiss et al., 1997; Fu et al., 2017). Increasing evidence suggests that abnormal changes in the function of dNK cells are closely related to adverse pregnancy outcomes such as preeclampsia and fetal growth restriction (Moretta et al., 2000a; Koopman et al., 2003). KIR2DL4 belongs to the killer cell immunoglobulin-like receptor (KIR) family of decidual NK cells, which interact with soluble HLA-G. The interaction between dNK inhibitory receptors (KIR2DL4, ILT-2) and their ligand HLA-G on invading trophoblasts allows the extensive remodeling of the maternal vasculature during the early weeks of pregnancy and inhibits the killing activity of NK cells to HLA-G positive trophoblasts (Yan et al., 2007; Ferreira et al., 2017). C-type lectin-like receptor NKG2A is also one of the inhibitory receptors of dNK cells, which has a higher affinity to HLA-E than CD94/NKG2C-activating receptor (El et al., 2009). Compared to normal pregnancy, the recurrent spontaneous abortion (RSA) group showed decreased expression of NKG2A on dNK (Sotnikova et al., 2014). Our previous studies have reported an excessive activation of dNK cells after *T. gondii* infection with imbalance between inhibitory receptors (NKG2A, KIR2DL4) and activating receptors NKG2D (Liu et al., 2013; Xu et al., 2013; Liu et al., 2014). Besides, IFN-γ-producing dNK cells counts were significantly increased following *T. gondii* infection (Liu et al., 2014; Zhang et al., 2015). However, the detailed mechanism of dNK cells dysfunction in *T. gondii* infection remains unknown and requires further study.

T cell immunoglobulin domain and mucin domain 3 (Tim-3) is known as an inhibitory molecule that transduces negative signals into T cell triggering apoptosis or exhaustion (Zhu et al., 2005). Recently, Tim-3 is found to be highly expressed on decidual immune cells, especially on dNK cells (Sun et al., 2016). Alteration of Tim-3 expression on dNK cells has been proven in several pregnancy failure and preeclampsia cases, identifying Tim-3 as a key mediator of dNK cells maintaining successful pregnancy (Miko et al., 2013; Meggyes et al., 2015; Li et al., 2017a). However, whether *T. gondii* infection could influence Tim-3 expression on dNK cells and subsequently lead to adverse pregnancy outcomes has not yet been investigated. In a previous experiment, we observed that *T. gondii* infection significantly induced downregulation of Tim-3 on dNK cells, and Tim-3^-/-^ pregnant mice exhibited more worse pregnancy outcomes compared with the WT group after infection. These findings indicated that Tim-3 expression may play an important role in adverse pregnancy outcomes by *T. gondii* infection. However, whether the change of Tim-3 expression after *T. gondii* infection leads to dNK cells dysfunction and then contributes to adverse pregnancy outcomes remains unclear. In the present study, Tim-3^-/-^ pregnant mice and Tim-3 neutralizing antibodies were used to fully elucidate the specific role of Tim-3 on dNK cells dysfunction following *T. gondii* infection.

## Materials and Methods

### Animal Models

C57BL/6 mice (WT mice; 6 to 8 week-old females and 8 to 10 week-old males) were purchased from Pengyue Laboratory Animal Technology Co., Ltd (Jinan, China). Tim-3-deficient (Tim-3^-/-^) C57BL/6 mice were obtained from Bioray Laboratories Inc (Shanghai, China). All mice were maintained in the specific pathogen-free animal house of Binzhou Medical University at 22°C-26°C with 50%-60% humidity and a 12 h light/12 h dark cycle, with access to abundant sterilized water and food (Jiangsu Biological Engineering Co., Ltd., China). Female mice were housed five per cage, and male mice were housed one per cage for a week to facilitate acclimatization to new environment. Subsequently, females and males were housed in cages at a 2:1 overnight, and female mice with vaginal plugs (gestational day, gd 0) were segregated and randomized into uninfected, *T. gondii* infected, and *T. gondii* infected-Tim-3^-/-^ groups. At gd 7, the infected group was intraperitoneally injected with 400 tachyzoites (RH strain) resuspended in 200 μl PBS, and the uninfected mice were injected with the same amount of PBS into the abdominal cavity.

### Scanning Electron Microscopy (SEM)

Mice were sacrificed on gd 11. All fetuses were carefully removed and washed 5-6 times in phosphate buffer (0.1 M), and fixed in 2.5% phosphate buffered glutaraldehyde for 2 days at 4°C. Thereafter, immobilized fetuses were dehydrated using a graded ethanol series every 15 min in each step. The samples were dried by the critical point technique (Quorum K850), attached to specimen holders and coated with gold particles by ion sputter coater (Quorum Q150RS). All samples were placed on sample stages and observed with a scanning electron microscope (ZEISS EVO LS15) operated at 10 KV. All images were obtained using SmarSEM user interface software.

### Obtention of Tim-3^-/-^ Mice

Mouse tails were used to extract genomic DNA, and polymerase chain reaction (PCR) was conducted to synthesize cDNA. After initial denaturation for 3 min at 94**°**C, PCR was performed with 30 amplification cycles of denaturation for 30 s at 94**°**C, annealing for 30 s at 56**°**C, and extension for 60 s at 72**°**C, followed by a final extension for 8 min at 72**°**C and maintenance at 16**°**C. The primers for PCR amplification were as follows:

Tim-3-PCR-S, 5′-GGCTGGCTCAAACTCACTACA-3′; Tim-3-PCR-A, 5′-CGGACAATGATAACATGGAAA-3′. Then PCR products were sequenced (Shanghai Majorbio Bio-Pharm Technology Co., Ltd), and we distinguished the homozygotes from heterozygotes or WT mice by analyzing the DNA chromatogram. A sufficient number of Tim-3^-/-^ mice were obtained and bred for use in the present study.

### Preparation of *T. gondii* Tachyzoites (RH Strain)

HEp-2 cells were obtained from the Cell Research Institute of the Chinese Academy of Sciences (Shanghai, China) and were used to maintain *T. gondii* tachyzoites in minimum essential medium (MEM; Hyclone) containing 5% fetal bovine serum (FBS; Gibco) and 100 IU/mL penicillin/streptomycin (Sigma-Aldrich). HEp-2 cells were centrifuged at 1500 rpm (433×*g*) for 10 min after culture, and the clear supernatants were then centrifuged at 4000 rpm (3082×*g*) to purify the tachyzoites. MEM was used to resuspend the tachyzoites. Then tachyzoites were counted by Neubauer chamber and cultured with new HEp-2 cells. The experiment was carried out in BSL-2 laboratories. All the labwares, consumables, and liquids contaminated by the parasites were collected and sterilized by high-pressure sterilizer.

### Cell Preparation of Mice

Mice uteri and placentas were carefully dissected from pregnant mice at gd 11 and were washed twice in cold PBS. Tissues were then cut into small pieces and prepared for cell dispersion by GentleMACS dissociator (Miltenyi Biotech). Sterile nets (48 µm) were used to obtain single cell suspensions. Mononuclear cells were collected from the white film layer after Ficoll density gradient centrifugation in mouse lymphocyte separation medium (TBD Science), and were then analyzed with flow cytometry. The mice cadavers were stored in a -20°C freezer and disposed of by professional organizations.

### Collection of Human Clinical Sample

The sample collection for this study was approved by the Ethics Committee of Binzhou Medical University, and all voluntary abortions had occurred in the Department of Obstetrics and Gynecology of Yantai Affiliated Hospital of Binzhou Medical University and the Yantai Hospital of Traditional Chinese Medicine. Decidual tissues of first trimester (gestational age of 6 to 8 weeks) were obtained from healthy pregnant women, and any evidence of threatened abortion or any pregnancy complications during gestation was excluded. Among these patients, those with abnormal chromosome abortuses, anatomic abnormalities of uterus and cervical, endocrine, and metabolic disease (diabetes, hyperthyroidism, and hypothyroidism), infection of chlamydia and ureaplasma in cervical mucus, or positive for anticardiolipin antibodies were excluded. Tissues were immediately rinsed with sterile saline solution 5-8 times, and decidual tissues were collected in Dulbecco’s modified Eagle’s medium/high glucose medium (Hyclone) supplemented with 100 IU/mL penicillin/streptomycin (Sigma-Aldrich).

### Isolation of Human dNK Cells

Decidual samples were immediately washed 5-6 times in Roswell Park Memorial Institute (RPMI) medium to remove the blood and then cut into small pieces. Tissues were maintained in C tubes containing 0.1% collagenase type IV (Sigma-Aldrich) and 25 IU/mL DNase-I (Sigma-Aldrich). Single cell suspensions were obtained by GentleMACS dissociator (Miltenyi Biotech) and were filtered through 48 µm nylon mesh filters. Then the mononuclear cells were isolated *via* density gradient centrifugation using human lymphocyte separation medium (TBD Science) at 2000 rpm (771×*g*) for 20 min at 20°C according to the manufacturer’s instructions. dNK cells were washed once by PBS and purified using a human CD3 positive selection kit and CD56 positive selection kit (Stem Cell Science) according to the manufacturer’s instructions with >95% purity ensured for experiments. Approximately 1.5×10^6^ purified human CD3^-^CD56^+^ dNK cells were obtained and divided equally into uninfected, infected, and Tim-3-neutralized infected groups. CD3^-^CD56^+^ dNK cells were incubated with 10 μg/mL anti-Tim-3 monoclonal antibody mAb (Thermo Fisher Scientific) in the Tim-3-neutralized infected group for 2 h, following which *T. gondii* tachyzoites were added to the Tim-3-neutralized infected group and the infected group at a 2:1 ratio (*T. gondii*: cells). All samples were cultured in RPMI medium supplemented with 10% FBS (FBS, Gibco) and 100 IU/mL penicillin/streptomycin (Sigma-Aldrich) for 36 h at 37°C in a humidified 5% CO_2_ incubator.

### Flow Cytometry

For surface staining, the following fluorochrome-conjugated mAbs were used: Tim-3 (clone RMT3-23, Biolegend), CD122 (clone TM-b1), CD3e (clone 145-2C11), NKG2A (clone 16a11), NKG2D (clone CX5, all from Thermo Fisher Scientific) were used *in vivo*. Isolated murine decidual mononuclear cells were incubated with mAbs above at 4°C in the dark for 30 min and then washed with PBS. *In vitro*, cells were stained with human-specific mAbs CD3 (clone OKT3), Tim-3 (clone 8B. 2C12), NKG2D (clone 5C6, all from Thermo Fisher Scientific), KIR2DL4 (clone mAb33, Biolegend) and CD56 (clone HCD56, Biolegend). For staining of intracellular granules Perforin, GranzymeA and GranzymeB, cells were firstly surface stained, subsequently fixed and permeabilized in 1× Fix/Perm buffer (Thermo Fisher Scientific) for 25 min according to the manufacturer’s protocol. After washing with buffer, cells were incubated with anti-Perforin (clone ebioOMAK-D), GranzymeA (clone GzA-3G8.5), GranzymeB (clone NGZB, all from Thermo Fisher Scientific) mAbs for 40 min at 4°C in the dark and then were washed with buffer. Human specific mAb GranzymeA (clone CB9, Biolegend), GranzymeB (clone GB11, BD bioscience) and Perforin (clone dG9, BD bioscience) were also used *in vitro*. For analysis of the intracellular cytokines, cells were initially stimulated for 4 h with a leukocyte activation cocktail (BD bioscience). Thereafter, the cells were collected and incubated with surface staining mAbs at 4°C for 40 min in the dark and then washed with buffer. The cells were fixed and permeabilized in 1× Fix/Perm buffer (Thermo Fisher Scientific) for 30 min at 4°C according to the protocol. After being washed twice, cells were incubated with mouse-specific mAbs IL-10 (clone JES5-16E3, BD bioscience) and IFN-γ (clone XMG1.2, BD bioscience) or human-specific IL-10 (clone JES3-19F1, BD bioscience) and IFN-γ (clone 4S.B3, BD bioscience) at 4°C in the dark for 40 min and washed with buffer. Analysis was performed using a FACScanto™ II instrument (Becton Dickinson).

### Immunofluorescence

Purified human CD3^-^CD56^+^ dNK cells from uninfected, infected, and Tim-3-neutralized infected groups were air-dried onto Poly-L-lysine coated slides. After fixation in 4% paraformaldehyde for 30 min, slides were then blocked with goat serum for 1 h at room temperature. dNK cells were incubated overnight at 4°C with anti-Tim-3 (1/200, Abcam), anti-NKG2D (1/200, Abcam), anti-GranzymeA, anti-GranzymeB and anti-Perforin (1/200, all from Proteintech) or for 45 min with anti-CD56 (clone HCD56, Biolegend) and anti-KIR2DL4 (clone mAb33, Biolegend) antibody. After being washed three times with PBS, cells were then incubated with appropriate concentrations of secondary antibodies for 1 h at 37°C. Cy3 rabbit anti-goat IgM (1/500, Bioss) was used as the secondary antibody for anti-Tim-3 antibody, and Cy3 donkey anti-rabbit IgG (1/500, Bioss) was used as secondary antibody for anti-NKG2D, anti-GranzymeA, anti-GranzymeB and anti-Perforin. Subsequently, dNK cells were stained with the DAPI (nucleic acid stain 4’,6-diamidino-2-phenylindole) for 15 min and washed 3 times with PBS. Finally, confocal microscopy of cells was performed using Zeiss LSM880.

### Western Blot Analysis

CD3^-^CD56^+^ dNK cells from the three groups were incubated for 36 h before harvesting. Equal amounts of protein from total-cell lysates were separated by 12% SDS-PAGE (Beyotime) and transferred onto polyvinylidene fluoride (PVDF) membranes (Millipore). The membranes were then blocked at room temperature for 2.5 h in 7% nonfat dry milk in TBS-T buffer. Membranes were incubated with gentle rocking 1.5 h at room temperature with primary antibodies for Tim-3 (1/2000, Proteintech), GranzymeB (1/2000, Abcam), Perforin (1/1000, Proteintech), GranzymeA (1/600, Proteintech), PI3K (1/500, Proteintech), AKT (1/500, SAB), pAKT (1/500, SAB), STAT1 (1/500, Proteintech), STAT3 (1/600, Proteintech), pSTAT1 (1/500, Abcam), pSTAT3 (1/600, Abcam), IL-10 (1/500, Abcam), IFN-γ (1/500, Proteintech) and GAPDH (1/40000, Proteintech) as a loading control. Membranes were washed with TBS-T 5 times for 10 min each, then incubated with the appropriate secondary antibody for 2 h at room temperature. Then immune complex was visualized with an enhanced chemiluminescence (ECL) detection kit (F. Hoffmann-La Roche, Ltd., Switzerland). Protein expression levels were determined by Image J software (Rawak Software, Inc., Germany).

### Histopathology

Mouse placentas and uteri of gd 11 were removed after treatments and washed thrice. After immediate fixation in 4% paraformaldehyde, tissues were washed in running water and placed in a graded ethanol and then were paraffin embedded using standard methods. Paraffin sections were cut to a thickness of 5 µm and stained with hematoxylin and eosin dye (H&E; Shanghai Novland Co., Ltd., China) according to the manufacturer’s instructions. Images of paraffin embedded sections were recorded at 40 × magnification and are presented with 50 μm scale bars.

### Statistical Analysis

Data are presented as the means ± SD. Statistical analyses were performed using the GraphPad Prism 5 Statistics software package. Unpaired *t* tests were used to identify differences. *p* **<** 0.05 was regarded as significant and *p* **<** 0.01 was considered as extremely significant.

## Results

### Tim-3^-/-^ Pregnant Mice Exhibited More Worse Pregnancy Outcomes Than WT Pregnant Mice After *T. gondii* Infection

**Figure 1:**
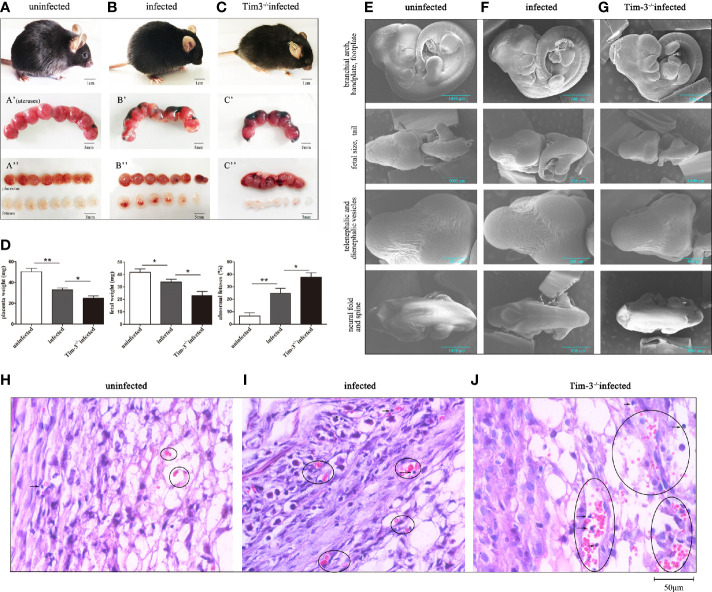
The impact of Tim-3 on adverse pregnancy outcomes caused by *T. gondii* infection in mice. **(A)** Uninfected pregnant mice were in good condition, the placentas and fetuses developed normally. **(B)** *T. gondii* infected pregnant mice exhibited spiritual malaise, with inflammatory hyperemia of placentas and fetuses. **(C)** *T. gondii* infected Tim-3^-/-^ pregnant mice had sluggish responses, their fetuses were smaller and shapeless. Scale bar of A, B, C was 1 cm. Scale bar of A’, A’’, B’, B’’, C’ and C’’ were 5 mm. **(D)** The weights of placentas and fetuses, and fetal abnormalities rates were calculated in uninfected, infected, Tim-3^-/-^ infected mice. The abnormal fetuses included stillbirths and resorption sites. The rates of abnormal fetus were calculated as the ratio of stillbirths and resorption sites to the total number of implantation sites. (Data are presented as means ± SD. N=10 mice per group, *p < 0.05, **p < 0.01, by the unpaired *t*-test). **(E)** Scanning electron microscopy images of fetuses from uninfected mice, the fetuses were healthy and well developed. **(F)** Fetuses of infected mice exhibited delayed hand-plates and foot-plates development. **(G)** Fetuses of infected Tim-3^-/-^ mice displayed obvious intrauterine growth restriction in head, body size, hand-plates and foot-plates development. Scale bar, 1000µm and 600µm. **(H-J)** Hematoxylin and eosin (H & E) staining of uninfected, infected and Tim-3^-/-^ infected mouse placentas. Obvious hemorrhaging and lymphocytes infiltration were showed by black circles. Scale bar, 50µm.

All mice were sacrificed and dissected on gd 11 to observe the pregnancy outcomes among three groups. We observed abnormal pregnancy outcomes caused by *T. gondii* infection, with erected fur, spiritual malaise, and inflammatory hyperemia of placentas in infected mice compared with the uninfected group (**Figures 1A, B**). Compared with infected WT mice, *T. gondii* infected Tim-3^-/-^ pregnant mice had sluggish responses, along with trembling and significant placental bleeding (**Figures 1B, C**). The fetuses of the infected WT group exhibited abnormal bleeding manifestations as well as a higher rate of abnormalities than those of the uninfected group (**Figures 1A, B**); in addition, decreased fetal and placental weights were observed in the fetuses of infected WT mice (**Figure 1D**). Fetuses of the infected Tim-3^-/-^ pregnant group were almost shapeless and smaller, with distinct developmental delays compared with infected WT fetuses (**Figures 1B, C**). The worse abnormal pregnancy outcomes in infected Tim-3^-/-^ pregnant mice were based on the decrease in fetal and placental weights and higher rate of abnormal fetuses in comparison with infected WT group (**Figure 1D**). We further observed the intrauterine development of embryos of three groups by scanning electron microscopy (SEM). Fetuses from the Tim-3^-/-^ infected mice had the smallest size among the three groups (**Figures 1E–G**), which is in line with our previous observations of fetal weight. Caused by infection of *T. gondii*, the fetuses in infected WT group exhibited smaller body and delayed formed paddle-shaped handplate and footplate (**Figures 1E, F**). The spinal development of infected fetuses was not as good as that of normal fetuses (**Figures 1E, F**). And the physical condition of the Tim-3^-/-^ infected fetuses was even worse, which was evidently indicated by their significantly smaller bodies, rarely formed paddle-shaped handplate and footplate, as well as smaller size of head compared with the findings in infected WT mice (**Figures 1F, G**). Paraffin embedded sections showed increased lymphocyte infiltration of the WT group placenta caused by *T. gondii* infection, and more severe damages in placentas of infected Tim-3^-/-^ group were found than WT infected mice, as evidenced by obvious hemorrhage and lymphocyte infiltration (**Figures 1H–J**). We found no differences in pregnancy outcomes between uninfected Tim-3^-/-^ mice and WT mice.

### Tim-3 Expression on dNK Cells Decreased After *T. gondii* Infection

**Figure 2:**
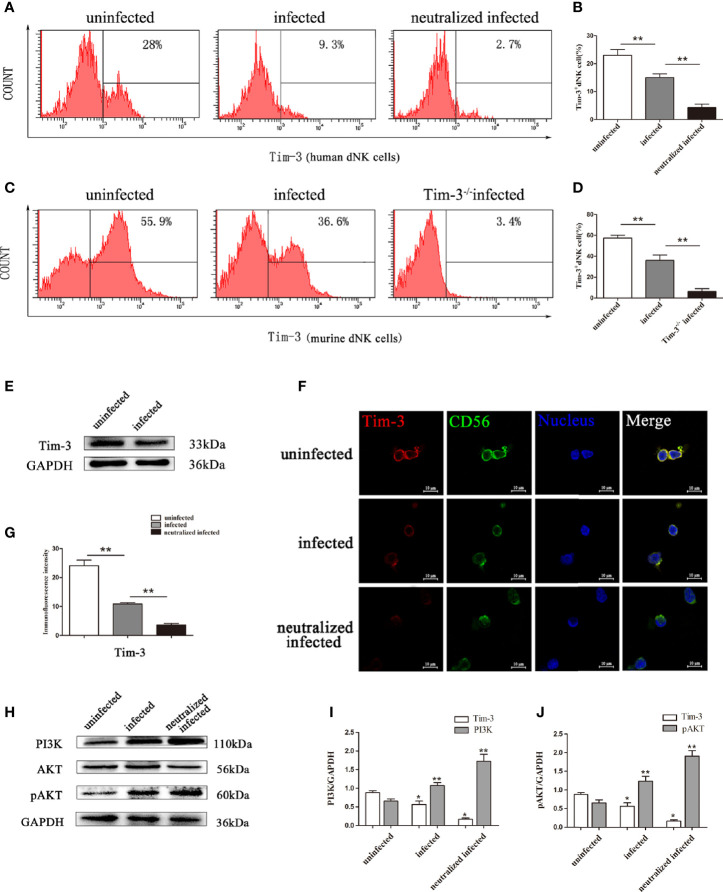
Changes of Tim-3 expressions on dNK cells due to *T. gondii* infection. **(A)** Expression levels of Tim-3 on human CD3^-^CD56^bright^ dNK cells in uninfected, infected, and anti-Tim-3 neutralized infected groups using flow cytometry. **(B)** Histograms analysis of Tim-3 expression changes on human dNK cells in the three groups. (Means ± SD; N=6 human samples per group, *p < 0.05, **p < 0.01, by unpaired *t*-test). **(C)** Expression of Tim-3 on mice CD3^-^CD122^+^ dNK cells in uninfected, infected, and Tim-3^-/-^ mice infected groups were detected by flow cytometry. **(D)** Histograms analysis of Tim-3 expression changes on mice dNK cells in uninfected, infected, and Tim-3^-/-^ mice infected groups. (Means ± SD, N=8 mice per group, *p < 0.05, **p < 0.01, by unpaired *t*-test). **(E)** Western blot analysis of Tim-3 expression in uninfected human dNK cells and infected groups. **(F)** Immunofluorescent photographs of Tim-3 (red), CD56 (green) expression on the purified human CD3^-^CD56^bright^ dNK cells from uninfected, infected, and Tim-3-neutralized and infected groups. The 4 ‘, 6-diamidino-2-phenylindole were used to stain nuclei (blue). Scale bar, 10µm. **(G)** Histograms analysis of Tim-3 expressions changes on human dNK cells by immunofluorescent intensity among three groups. **(H)** Representative depictions of Tim-3 downstream molecules PI3K, AKT and pAKT expressions in uninfected, infected, and Tim-3-neutralized infected human dNK cells by western blot. **(I, J)** The histograms analysis of Tim-3, PI3K and pAKT among three groups. (Means ± SD; N=6 human samples per group, *p < 0.05, **p < 0.01, by unpaired *t*-test).

To investigate the role of Tim-3 expression during *T. gondii* infection, Tim-3 expression levels on dNK cells in decidual lymphocytes and murine decidual lymphocytes were analyzed by flow cytometry, western blot, and confocal laser-scanning microscope. The results demonstrated that Tim-3 expression levels on dNK cells were significantly decreased both in human dNK cells (**Figures 2A, B, E–G**) and in mice dNK cells (**Figures 2C, D**) after *T. gondii* infection compared with the uninfected group. In addition, the downstream molecules modulated by Tim-3 were detected through western blot analysis. Results showed that PI3K and pAKT were all increased with *T. gondii* infection compared with the uninfected group, and were further up-regulated in infected Tim-3-neutralized human dNK cells (**Figure 2H**), which confirmed the PI3K and pAKT as downstream molecules regulated by Tim-3 (**Figures 2H-J**).

### Down-Regulation of Tim-3 Expression by *T. gondii* Infection Was Associated With the Balance Between Inhibitory and Activating Receptors on dNK Cells

**Figure 3:**
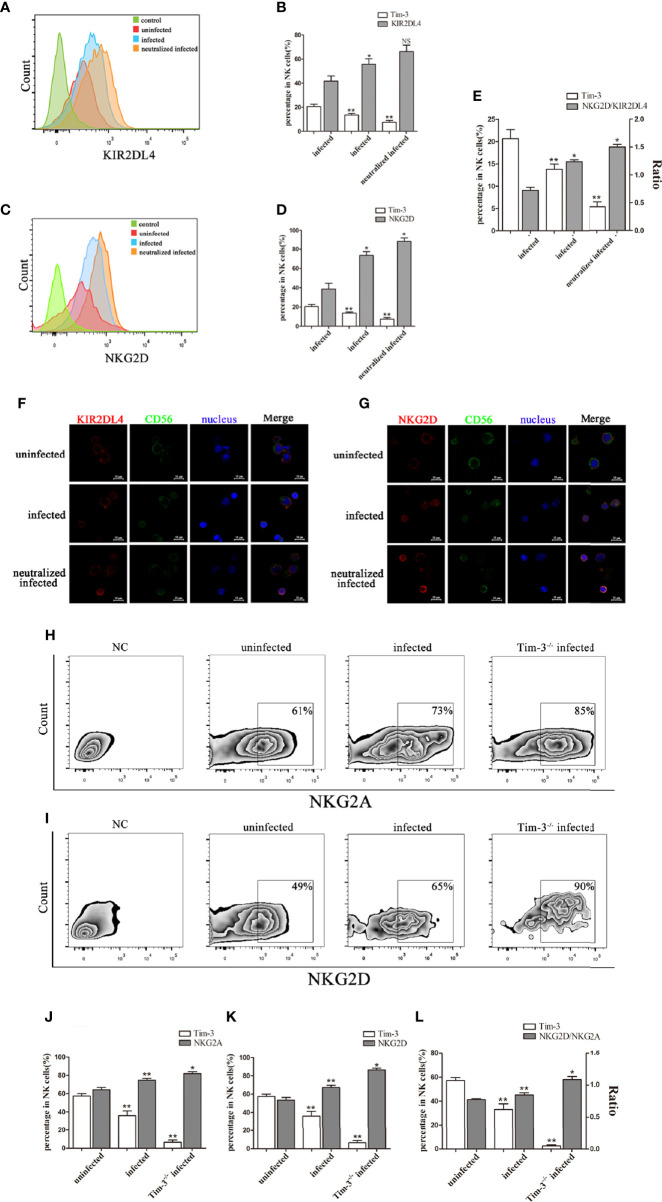
Reduction of Tim-3 expression by *T. gondii* infection is associated with the imbalance of inhibitory and activating receptors on dNK cells. **(A, C)** Flow cytometry analysis revealed that expression of Tim-3, KIR2DL4, NKG2D on human dNK cells were altered in uninfected, infected, and anti-Tim-3 neutralized infected cells from patients who underwent induced abortion. **(B, D, E)** Statistical analysis of Tim-3, KIR2DL4 and NKG2D expression and ratios of NKG2D/KIR2DL4 on human dNK cells among three groups. (Means ± SD; N=6 human samples per group, *p < 0.05, **p < 0.01, by unpaired *t*-test. NS, Not Statistically Significant) **(F, G)** Representative immunofluorescent photographs of KIR2DL4 (red), CD56 (green) and NKG2D (red) expression in uninfected, infected, and anti-Tim-3 neutralized infected human dNK cells. Nuclei (blue) were stained by 4’, 6-diamidino-2-phenylindole. Representative expression changes of inhibitory receptor NKG2A. **(H, J)** and activating receptor NKG2D **(I, K)** on mice dNK cells from uninfected, infected and Tim-3^-/-^ infected mice. **(L)** Histogram analysis of Tim-3 and ratios of NKG2D/NKG2A in three groups. (Means ± SD; N=8 mice per group, *in vivo*, *p < 0.05, **p < 0.01, by unpaired *t*-test).

We then detected the dNK cells receptors both *in vitro* and *in vivo*. Results found that up-regulation of inhibitory receptor KIR2DL4 (*in vitro*, **Figures 3A, B, F**), NKG2A (*in vivo*, **Figures 3H, J**) and activating receptor NKG2D (*in vitro*, **Figures 3C, D, G**; *in vivo*, **Figures 3I, K**), with high ratios of NKG2D/KIR2DL4 and NKG2D/NKG2A on dNK cells in the *T. gondii* infected group (**Figures 3E, L**). Besides, anti-Tim-3 neutralized infected human dNK cells and *T. gondii* infected Tim-3^-/-^ pregnant mice were used to further explore the effect of Tim-3 down-regulation on the balance between inhibitory and activating receptors. In the Tim-3 neutralized infected group, NKG2D expression was up-regulated and the imbalance of NKG2D/KIR2DL4 on dNK cells was further aggravated compared with infected cells (**Figures 3A-G**). Moreover, the expression of inhibitory receptor NKG2A and activating receptor NKG2D on murine dNK cells increased in infected Tim-3^-/-^ mice; however, the ratio of NKG2D/NKG2A was significantly higher than that of WT infected mice (**Figures 3H-L**).

### Tim-3 Down-Regulation by *T. gondii* Infection Altered the GranzymeA, GranzymeB, and Perforin Expressions of dNK Cells

**Figure 4:**
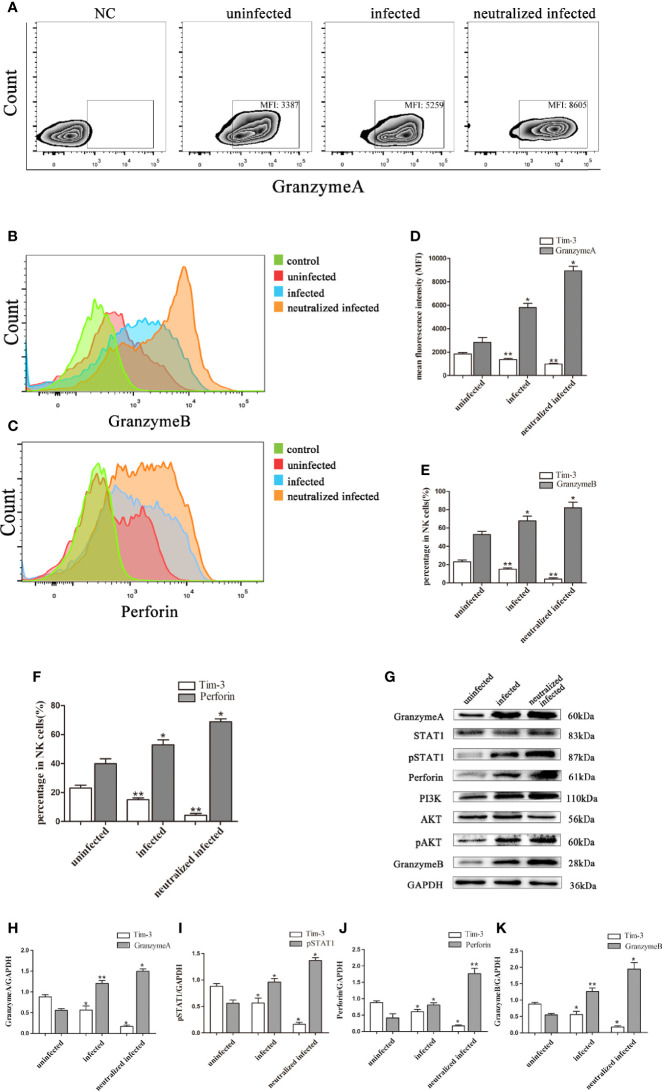
Tim-3 down-regulation by *T. gondii* infection altered the GranzymeA, GranzymeB and Perforin expressions of human dNK cells. **(A-F)** Representative flow cytometry and histograms analysis of GranzymeA **(A, D)**, GranzymeB **(B, E)** and Perforin **(C, F)** levels of human dNK cells in the uninfected, infected, and neutralized infected groups. (Means ± SD; N=6 human samples per group, *p < 0.05, **p < 0.01, by unpaired *t*-test). **(G)** Western blot analysis of signaling pathway of Perforin and GranzymeB. Since GranzymeB is also modulated by PI3K-AKT signaling pathway and the experiments were performed at the same time, the data of PI3K, AKT and pAKT in **Figure 4G** were reused from the data of **Figure 2H**. **(H-K)** Histograms analysis of GranzymeA, pSTAT1, Perforin and GranzymeB in uninfected, infected, and anti-Tim-3 neutralized infected groups. (Means ± SD; N=6 human samples per group, *p < 0.05, **p < 0.01, by unpaired *t*-test).

**Figure 5:**
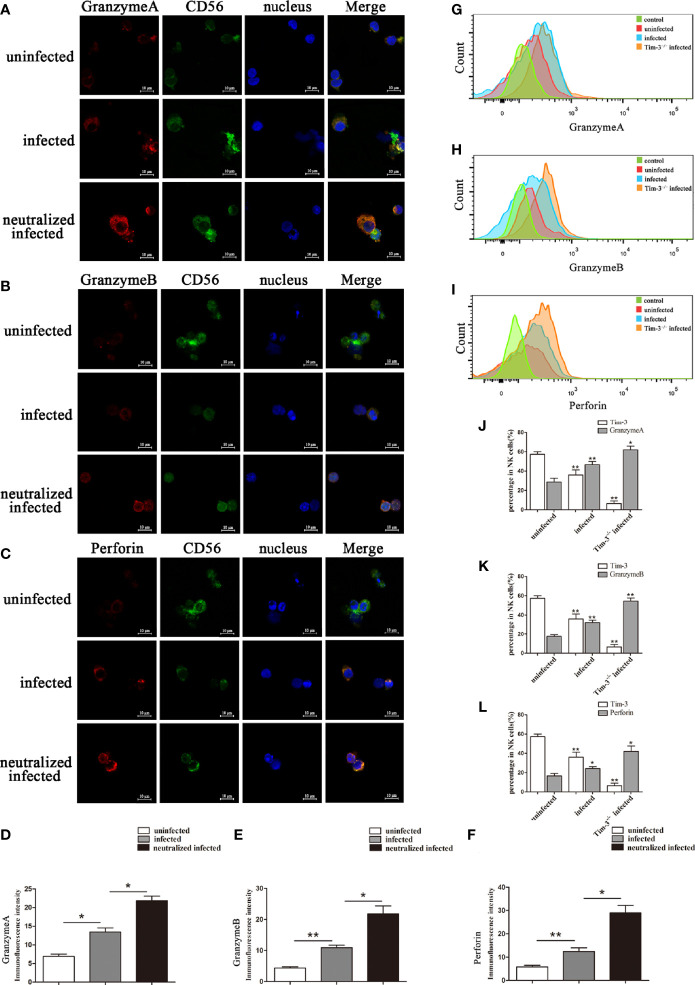
Representative confocal micrographs of GranzymeA, GranzymeB and Perforin in human dNK cells and flow cytometry analysis of mice dNK cells. **(A-F)** Representative immunofluorescent photographs and immunofluorescent intensity analysis of GranzymeA (red), CD56 (green), GranzymeB (red) and Perforin (red) expression in uninfected, infected, and anti-Tim-3 neutralized infected huamn dNK cells. Nuclei were blue and stained using 4’, 6-diamidino-2-phenylindole. Scale bar, 10µm. (Means ± SD; N=6 human samples per group, *p < 0.05, **p < 0.01, by unpaired *t*-test). **(G-L)** In uninfected, infected and Tim-3^-/-^ infected mice, dNK cells expression of GranzymeA **(G, J)**, GranzymeB **(H, K)** and Perforin **(I, L)** levels were analyzed by flow cytometry and compared by unpaired *t*-test. (Means ± SD; N=8 mice per group, *p < 0.05, **p < 0.01, by unpaired *t*-test).

Granules production was analyzed in the three groups to explore the impact of Tim-3 in modulating dNK cells function. *In vitro*, the GranzymeA, GranzymeB and Perforin expressions of dNK cells were analyzed by flow cytometry, western blot, and immunofluorescence respectively (**Figures 4A-K**, **5A-F**). The data revealed that the expressions of GranzymeA, GranzymeB and Perforin in CD3^-^CD56^+^ NK cells were increased markedly with *T. gondii* infection compared to the uninfected group (**Figures 4A-K**, **5A-F**). Interestingly, the GranzymeA, GranzymeB, and Perforin production of human dNK in Tim-3 neutralized group were all distinctly higher than those of infected dNK cells (**Figures 4A-K**, **5A-F**). In accordance with the result *in vitro*, the expression levels of GranzymeA, GranzymeB and Perforin in mice dNK cells increased after *T. gondii* infection, and further increased in infected Tim-3^-/-^ mice (**Figures 5G-L**).

### Down-Regulation of Tim-3 by *T. gondii* Infection Affected IL-10 and IFN-γ Production in dNK Cells

**Figure 6:**
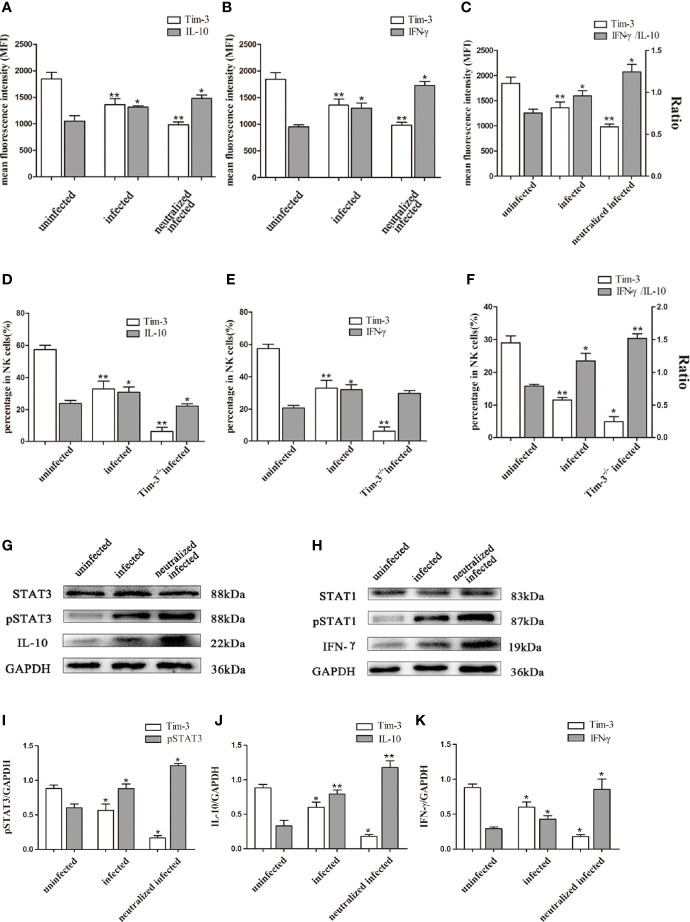
Down-regulation of Tim-3 by *T. gondii* infection affected IL-10 and IFN-γ secretion in dNK cells. **(A-C)** The human dNK cells expression levels of Tim-3, IL-10 **(A)**, IFN-γ **(B)** and IFN-γ/IL-10 ratios **(C)** among uninfected, infected, and anti-Tim-3 neutralized infected groups were detected by flow cytometry. **(D-F)** Tim-3, IL-10 **(D)**, IFN-γ **(E)** expression changes and ratios of IFN-γ/IL-10 **(F)** in uninfected, infected, and Tim-3^-/-^ infected mice dNK cells were showed by flow cytometry. (Means ± SD; N=6 human samples per group, *in vitro*; N=8 mice per group, *in vivo*, *p < 0.05, **p < 0.01, by unpaired *t*-test). **(G)** STAT3 and pSTAT3 involved in the regulation of IL-10 secretion were detected by western blot among uninfected, infected, and anti-Tim-3 neutralized infected groups. **(H)** STAT1 and pSTAT1 which were associated with IFN-γ expression in dNK cells were detected by western blot in uninfected, infected, and anti-Tim-3 neutralized infected groups. The data of STAT1 and pSTAT1 in **Figure 5H** reused the data in **Figure 4G** for Perforin and IFN-γshares the same upstream molecules STAT1 and pSTAT1, and the detection was conducted simultaneously. **(I-K)** Histograms analysis of pSTAT3, IL-10 and IFN-γ in uninfected, infected, and anti-Tim-3 neutralized infected groups. (Means ± SD; N=6 human samples per group, *p < 0.05, **p < 0.01, by unpaired *t*-test).

In the present study, flow cytometry and western blot were used to elucidate the relationship between Tim-3 expression and cytokine production of dNK cells. Results showed that the IL-10 and IFN-γ secretion increased in infected Tim-3-neutralized human dNK cells compared with that of the infected group (**Figures 6A, B, G, J, K**). While the ratio of IFN-γ/IL-10 were higher in infected Tim-3-neutralized cells than that of infected group (**Figure 6C**). *In vivo*, IL-10 and IFN-γ expression of dNK cells were both decreased in Tim-3^-/-^ infected mice (**Figures 6D, E**) but IFN-γ/IL-10 ratios were higher than that of infected WT mice (**Figure 6F**).

To investigate how the mechanism of Tim-3 regulates the expression of IL-10 and IFN-γ in dNK cells, the expression of key molecules of JAK-STAT signaling pathway were examined by western blot. The pSTAT3 expression increased following *T. gondii* infection and were further up-regulated in infected Tim-3-neutralized human dNK cells. This result suggested that enhanced pSTAT3 may take part in the change of IL-10 induced by down-regulated Tim-3 after *T. gondii* infection (**Figure 6G, I, J**). In addition, the pSTAT1 expression related to IFN-γ synthesis was elevated with *T. gondii* infection and further increased in infected Tim-3-neutralized human dNK cells (**Figure 6H, K**).

## Discussion

As one of the common causative agents of prenatal infections during pregnancy, *T. gondii* infection evokes a strong immune response in maternal-fetal interface and leads to adverse pregnancy outcomes (Wong and Remington, 1994; Li et al., 2017b). Our previous studies have reported that the dysfunctions of decidual immune cells such as dNK cells, macrophages, Tregs, and DCs in response to *T. gondii* infection result in abnormal pregnancy outcomes (Zhang et al., 2012a; Li et al., 2017b; Xu et al., 2017). Our results also demonstrated that *T. gondii* infection induces an imbalance of dNK receptors and abnormal cytokines secretion, which excessively activated dNK cells and further led to adverse outcomes of pregnancy (Liu et al., 2013; Xu et al., 2013). However, the detailed mechanism underlying *T. gondii* infection-induced dNK dysfunction is still unclear. The newly defined immunosuppressive molecule Tim-3 has been found to be consistently expressed on dNK cells and reportedly modulates the function of dNK cells (Li et al., 2016; Li et al., 2017a). Emerging evidence has demonstrated that Tim-3 expression plays an important role in sustaining normal pregnancy (Zhan et al., 2018). Researchers have found that the changes in Tim-3 expression on dNK cells result in pregnancy complications such as preeclampsia and miscarriage (Meggyes et al., 2015; Sun et al., 2016; Li et al., 2017a). However, the effect of *T. gondii* infection on Tim-3 expression of dNK cells requires further investigation. Interestingly, the results of the present study showed that the Tim-3 expression on dNK cells was significantly decreased after *T. gondii* infection. To explore the mechanism of Tim-3 down-regulation on dNK cells dysfunction in abnormal pregnancy caused by *T. gondii* infection, Tim-3^-/-^ infected pregnant mice models were successfully established. The results revealed that Tim-3^-/-^ infected pregnant mice exhibited far worse pregnancy outcomes compared with infected WT mice. This finding suggested that Tim-3 expression may play an important role in abnormal pregnancy outcomes involving *T. gondii* infection. In the present study, *in vitro* and *in vivo* experiments were performed to further uncover the mechanism of Tim-3 in modulating dNK cells function during *T. gondii* infection. Previous studies have proved that the function of dNK cells was precisely regulated by the balance of activating and inhibitory receptors (Moretta et al., 2000b). KIR2DL4 and NKG2A are both inhibitory receptors and sustain the tolerance function of dNK cells in pregnancy, by antagonizing the activating function of NKG2D (Rouas-Freiss et al., 1997; Moretta et al., 2000a; Moretta et al., 2000b). Recently, research has demonstrated that the over-expression of NKG2D elicited an enhanced local cytotoxic response by NK cells which was associated with preeclampsia (Liu et al., 2017; Vinnars et al., 2018). In our previous research, we observed that *T. gondii* infection disturbed the balance of NKG2D and KIR2DL4 (NKG2A *in vivo*) on dNK cells, which is an important aspect of dNK cells dysfunction in *T. gondii*-mediated adverse pregnancy (Liu et al., 2013; Xu et al., 2013). However, the specific mechanism of the imbalanced receptor expression has not yet been investigated. In this study, human dNK cells were purified and cultured with *T. gondii*, and Tim-3 was blocked with Tim-3 neutralizing antibody. The result indicated that NKG2D/KIR2DL4 ratios of dNK cells were considerably higher in Tim-3-neutralized group following *T. gondii* infection than that of the infected group. This demonstrated that the down-regulation of Tim-3 expression on dNK cells after *T. gondii* infection could result in an imbalance of inhibitory and activating receptors. In accordance with the results *in vitro*, the present study declared that the NKG2D/NKG2A ratio of dNK cells was increased after *T. gondii* infection and further enhanced in Tim-3^-/-^ infected mice. Considering the different abnormal pregnancy outcomes between Tim-3^-/-^ infected mice and WT infected mice, we could deduce that the down-regulation of Tim-3 expression on dNK cells after *T. gondii* infection caused an imbalance between inhibitory and activating receptors, thereby contributing to abnormal pregnancy outcomes.

The cytotoxic molecules GranzymeA, GranzymeB and Perforin are constitutively produced by NK cells (Trapani et al., 1998; Shirshev et al., 2017; Gulic et al., 2018). Researchers have found that dysfunctional dNK cells release excessive GranzymeB and Perforin which induce pregnancy failure (Trapani et al., 1998; Gulic et al., 2018). And our precious study revealed that *T. gondii* infection could up-regulate cytotoxic production such as GranzymeA, GranzymeB and Perforin at maternal-fetal interface (Xu et al., 2017; Zhang et al., 2018). In this study, the increased expression of Granzymes and Perforin in dNK cells were demonstrated after *T. gondii* infection *in vivo* and *in vitro*. Furthermore, results showed significantly higher expression of GranzymeA, GranzymeB, and Perforin in infected Tim-3-neutralized dNK cells and Tim-3^-/-^ infected mice than that of infected dNK cells, which demonstrated that the reduction of Tim-3 on dNK cells further enhanced GranzymeA, GranzymeB, and Perforin production. According to the severe pregnancy outcomes of infected Tim-3^-/-^ mice, it was concluded that decreased Tim-3 on dNK cells caused by *T. gondii* infection led to excessive cytotoxic production by dNK cells, which participate in the development of pregnancy failure.

Besides, dNK cells can also secrete many cytokines such as IL-10 and IFN-γ that participate in maternal-fetal tolerance (Trundley and Moffett, 2004). Dysregulation of IL-10 has been widely confirmed in pregnancy complications such as preeclampsia, recurrent spontaneous abortion, and preterm birth (Murphy et al., 2005; Yamada et al., 2005; Makris et al., 2006). It is known that IL-10 in dNK cells plays a key role in sustaining maternal-fetal tolerance function of dDCs (Miyazaki et al., 2003). Coversely, excessive IFN-γ secretion of dNK could provoke pregnancy failure by inducing aberrant recruitment of CD49b^+^ NK cells (Li et al., 2014). Additionally, emerging evidence has proven that over-expression of IFN-γ affects immune-regulation in maternal-fetal interface and leads to recurrent pregnancy loss and implantation failure (Wilczyński et al., 2003). Our previous study has revealed that the *T. gondii* infection induced excessive secretion of IFN-γ by dNK cells (Zhang et al., 2015). And we found that the IFN-γ/IL-10 ratios in placenta supernatant from *T. gondii* infected mice were higher than normal pregnant mice (Zhang et al., 2012b). In current research, the IFN-γ/IL-10 ratios in dNK cell were obviously increased after *T. gondii* infection both *in vivo* and *in vitro*, and further up-regulated in infected Tim-3-neutralized dNK and infected Tim-3^-/-^ mice. This suggested that the decrease of Tim-3 on dNK cells could up-regulate ratios of IFN-γ/IL-10 and impact the tolerance function of dNK cells. On the basis of these findings, *T. gondii* infection-induced down-regulation of Tim-3 could result in dysfunction of dNK cells due to the up-regulation of IFN-γ/IL-10 ratio, and finally contribute to adverse pregnancy outcomes.

However, the detailed mechanism of Tim-3 modulating the cytokines (IFN-γ, IL-10) synthesis, Perforin, and GranzymeB production had not been confirmed during *T. gondii* infection. AKT in NK cells has been identified as a downstream molecular modulated by Tim-3 (Li et al., 2017a). Also, AKT is proved as a key molecular in PI3K-AKT-mTOR pathway modulating the GranzymeB expression and IFN-γ production by NK cells (Nandagopal et al., 2014; Li et al., 2017a). To further explore the pathways *via* which Tim-3 regulates cytokines secretion and cytotoxic production of dNK cells, the AKT, pAKT, PI3K, GranzymeB, IFN-γ expression in infected human dNK cells and in Tim-3 neutralized human dNK cells were analyzed with western blot. The results demonstrated that *T. gondii* infection increased the expression of PI3K and pAKT, leading to significant up-regulation of GranzymeB and IFN-γ expression, and the PI3K and pAKT being further up-regulated with significantly higher expression of GranzymeB and IFN-γ in Tim-3 neutralized infected dNK cells. Based on these data, it was revealed that *T. gondii*-mediated Tim-3 reduction could lead to activation of PI3K-AKT signaling pathway and further enhanced the production of GranzymeB and IFN-γ in dNK cells. In addition, pSTAT1 is also considered an important regulator of IFN-γ and Perforin production by NK cells (Miyagi et al., 2007). The phophorylation of STAT1 could up-regulate the Perforin and IFN-γ production of NK cells (Vargas-Hernández et al., 2018). Besides, pSTAT3 is considered an important regulator during the synthesis of IL-10 (Braunschweig et al., 2011). Our western blot results showed that the expression levels of pSTAT1 and pSTAT3 were all up-regulated in induced *T. gondii* infection and were even higher in infected Tim-3 neutralized dNK cells. Combining the western blot analysis of IFN-γ, Perforin, IL-10, pSTAT1, and pSTAT3, we confirmed that the inflammatory effector (IFN-γ, Perforin) and anti-inflammatory factor IL-10 were separately correlated with phosphorylation level of STAT1 and pSTAT3. Many effector proteins are injected into host cells during *T. gondii* infection and modulate cell functions (Koshy et al., 2012). *T. gondii* was found to activate the host STAT (signal transducer and activator of transcription) signaling pathway by enhancing phosphorylation level. And our research confirmed that phosphorylation of STAT1 and STAT3 of dNK cells was increased after *T. gondii* infection. Many studies proved that parasite rhoptry protein ROP16 participated in modulating STAT3 and STAT6 phosphorylation of infected host cells (Yamamoto et al., 2009; Butcher et al., 2011; Hunter and Sibley, 2012). However, we found that pSTAT1 and pSTAT3 expression was further increased in infected Tim-3 neutralized dNK cells, which suggests that Tim-3 as an important modulator of pSTAT1 and pSTAT3. This result indicated that the reduction of Tim-3 caused by *T. gondii* infection could enhance pSTAT1 and pSTAT3 expression, then leading to changes in cytokines (IL-10, IFN-γ) secretion and Perforin production. Upon invasion, *T. gondii* parasites down-regulated the Tim-3 expression of dNK cells, and the reduction of Tim-3 further modulating the cytokines and granules production by activating the PI3K-AKT and JAK-STAT signaling pathway.

In summary, *T. gondii* infection-mediated Tim-3 reduction resulted in the imbalance of dNK cells receptors, cytotoxic granules overproduction, and abnormal cytokine secretion through activating PI3K-AKT and JAK-STAT signaling pathways, leading to the dysfunction of dNK cells which participated in the development of abnormal pregnancy outcomes. The present investigation also offers a new understanding about the immune molecular mechanisms underlying aberrant pregnancy outcomes caused by *T. gondii* infection.

## Data Availability Statement

The raw data supporting the conclusions of this article will be made available by the authors, without undue reservation.

## Ethics Statement

The studies involving human participants were reviewed and approved by the Ethics Committee of Binzhou Medical University (Shandong, China) for sample collection from women undergoing voluntary termination of gestation. The patients/participants provided their written informed consent to participate in this study. The animal study was reviewed and approved by the Ethics Committee of Binzhou Medical University (Shandong, China). This study was conducted in strict accordance with the requirements of the Guide for the Care and Use of Laboratory Animals of Binzhou Medical University. All experimental procedures were performed under sodium pentobarbital anesthesia to minimize the suffering of laboratory animals.

## Author Contributions

TL, LC, XX, and XH designed the experiments. LC, LR, and CY contributed to sample collection. TL, XL, HZ, and YJ analyzed the data. TL, LC, XX, and XH wrote the manuscript. TL, LC, XX, and XH edited the manuscript. XH was the corresponding author. All authors contributed to the article and approved the submitted version.

## Funding

This work was supported by funds from the National Natural Science Foundation of China (NO. 81871680, NO. 81702029, and NO. 81672049) and the Taishan Scholar Foundation of Shandong province (NO. ts201712066). The content of this manuscript in part has been presented at the 17^th^ International Congress of Immunology.

## Conflict of Interest

The authors declare that the research was conducted in the absence of any commercial or financial relationships that could be construed as a potential conflict of interest.
